# Blood Biomarkers of Late Pregnancy Exposure to Trihalomethanes in Drinking Water and Fetal Growth Measures and Gestational Age in a Chinese Cohort

**DOI:** 10.1289/ehp.1409234

**Published:** 2015-08-18

**Authors:** Wen-Cheng Cao, Qiang Zeng, Yan Luo, Hai-Xia Chen, Dong-Yue Miao, Li Li, Ying-Hui Cheng, Min Li, Fan Wang, Ling You, Yi-Xin Wang, Pan Yang, Wen-Qing Lu

**Affiliations:** 1 Department of Occupational and Environmental Health, School of Public Health, Tongji Medical College, Huazhong University of Science and Technology, Wuhan, Hubei, PR China; 2 Key Laboratory of Environment and Health, Ministry of Education & Ministry of Environmental Protection, and State Key Laboratory of Environment and Health (incubating), School of Public Health, Tongji Medical College, Huazhong University of Science and Technology, Wuhan, Hubei, PR China; 3 Department of Gynecology and Obstetrics, Wuhan No.1 Hospital, Wuhan, Hubei, PR China; 4 Department of Gynecology and Obstetrics, Xiaonan Maternal and Child Care Service Centre, Xiaogan, Hubei, PR China

## Abstract

**Background:**

Previous studies have suggested that elevated exposure to disinfection by-products (DBPs) in drinking water during gestation may result in adverse birth outcomes. However, the findings of these studies remain inconclusive.

**Objective:**

The purpose of our study was to examine the association between blood biomarkers of late pregnancy exposure to trihalomethanes (THMs) in drinking water and fetal growth and gestational age.

**Methods:**

We recruited 1,184 pregnant women between 2011 and 2013 in Wuhan and Xiaogan City, Hubei, China. Maternal blood THM concentrations, including chloroform (TCM), bromodichloromethane (BDCM), dibromochloromethane (DBCM), and bromoform (TBM), were measured as exposure biomarkers during late pregnancy. We estimated associations with gestational age and fetal growth indicators [birth weight, birth length, and small for gestational age (SGA)].

**Results:**

Total THMs (TTHMs; sum of TCM, BDCM, DBCM, and TBM) were associated with lower mean birth weight (–60.9 g; 95% CI: –116.2, –5.6 for the highest vs. lowest tertile; p for trend = 0.03), and BDCM and DBCM exposures were associated with smaller birth length (e.g., –0.20 cm; 95% CI: –0.37, –0.04 for the highest vs. lowest tertile of DBCM; p for trend = 0.02). SGA was increased in association with the second and third tertiles of TTHMs (OR = 2.91; 95% CI: 1.32, 6.42 and OR = 2.25; 95% CI: 1.01, 5.03; p for trend = 0.08).

**Conclusions:**

Our results suggested that elevated maternal THM exposure may adversely affect fetal growth.

**Citation:**

Cao WC, Zeng Q, Luo Y, Chen HX, Miao DY, Li L, Cheng YH, Li M, Wang F, You L, Wang YX, Yang P, Lu WQ. 2016. Blood biomarkers of late pregnancy exposure to trihalomethanes in drinking water and fetal growth measures and gestational age in a Chinese cohort. Environ Health Perspect 124:536–541; http://dx.doi.org/10.1289/ehp.1409234

## Introduction

Chlorine, due to its efficacy and cost-effectiveness, has been extensively used in the treatment of drinking water to reduce the risk of waterborne disease worldwide, including in China. However, chlorine and other disinfectants can react with natural organic and inorganic matter that occurs in water to form disinfection by-products (DBPs), which have been suggested to be potentially carcinogenic and to exert reproductive and developmental toxicities (Nieuwenhuijsen et al. 2010). Trihalomethanes (THMs) are the most abundant DBP class in drinking water; they include chloroform (TCM), bromodichloromethane (BDCM), dibromochloromethane (DBCM), and bromoform (TBM) (Nieuwenhuijsen et al. 2000). Widespread exposure to THMs can result from ingestion, inhalation, and dermal absorption during routine water-use activities such as drinking, washing, showering, bathing, and swimming. Based on the potential adverse health effects of exposure to DBPs, four THMs have been regulated in the European Union, the United States, and other countries (e.g., in Australia and China).

Toxicological studies have found that THMs may result in adverse reproductive effects. Exposure to TCM and DBCM through oral administration has been shown to cause fetal toxicity in rats including decreased body weight, body length, and survival rate (Ruddick et al. 1983). Exposure to BDCM at high doses has also been shown to cause pregnancy loss in rats (Bielmeier et al. 2001). A number of epidemiological studies have also examined the relationship between DBP exposure and adverse reproductive outcomes, including pregnancy loss, birth defects, and fetal growth restriction (Dodds et al. 2004; Grazuleviciene et al. 2013; Hoffman et al. 2008b; Infante-Rivard 2004; Savitz et al. 2006; Toledano et al. 2005); however, the findings of these studies are equivocal. One of the major limitations of previous studies is inaccurate exposure assessment (Nieuwenhuijsen et al. 2009). Most researchers took advantage of routinely collected measurements of THM concentrations in public water supplies as a surrogate of exposure, and some also combined data from water-use activities to estimate internal THM dose. However, these exposure assessments may result in misclassification of exposure by several factors: spatial and temporal variability of THMs in water systems, the contribution of different exposure routes, inter- and intra-individual variability in water usage (including residential mobility), and inter-and intra-individual physiological differences in absorption, distribution, metabolism, and excretion of the four THMs (Backer et al. 2000, 2008; Leavens et al. 2007).

Exposure biomarkers can represent integrative measures of all routes of exposure and provide an accurate exposure assessment for specific exposure windows. THM concentrations in blood and alveolar air samples have been measured to assess internal dose levels of THMs (Gordon et al. 2006; LaKind et al. 2010). Although the collection of breath samples is noninvasive, THM concentrations were generally undetectable before high levels of exposure (Weisel et al. 1999). In contrast, blood THM concentrations were generally more sensitive to low levels of exposure (Backer et al. 2000; Weisel et al. 1999). Although the elimination half-life of THMs in blood is short (minutes–hours), there are believed to be steady-state concentrations due to repeated and relatively consistent exposure to THMs (Blount et al. 2011). Several factors have been associated with blood THM levels including THM concentrations in water distribution systems, water-use activities (e.g., bathing/showering, swimming), personal sociodemographic characteristics (e.g., age, body mass index, education, household income), and genetic and physiological differences (Aggazzotti et al. 1998; Backer et al. 2008; Caro and Gallego 2007; Lynberg et al. 2001; Nuckols et al. 2005; Riederer et al. 2014; Rivera-Núñez et al. 2012; Zeng et al. 2014b).

We conducted a study in Wuhan and Xiaogan city, Hubei, China to investigate the relationships between exposure to drinking-water DBPs and birth outcomes. In our study, whole-blood THM levels were determined to assess the internal dose of THM exposure. To our knowledge, our study is the first to use THM levels in whole blood as exposure biomarkers to evaluate the effects of exposure to THMs in drinking water on birth outcomes.

## Methods

*Study participants.* We conducted a study in two contiguous cities, Wuhan and Xiaogan, Hubei, China. The water distribution systems in the two cities are supplied by surface water sources, and chlorine is used in the water treatment process. Women in late pregnancy during July 2011 to July 2012 in Wuhan and during October 2012 to December 2013 in Xiaogan were invited to participate in the study. The study was approved by the Ethics Committee of Tongji Medical College, and all participants provided written informed consent at the time of enrollment.

During the study period, a total of 997 and 750 pregnant women during late pregnancy (≥ 35 weeks) in Wuhan and Xiaogan agreed to participate in the study, respectively. Of them, 1,261 (72%) provided blood samples for analysis. We restricted our analyses to single gestation live infants, whose mothers lived in the local city for at least 1 year (*n* = 77 excluded participants), resulting in a total of 1,184 births.

*Questionnaires.* All participants took part in a face-to-face interview conducted by the trained investigators to complete a structured questionnaire on the first day of hospital admittance waiting for delivery. The questionnaire included demographics, lifestyle, occupational exposures during pregnancy, gravidity, parity, case history, and routine water-use activities. Data regarding water-use activities included source of drinking water, use of boiled water and filtered water, the total volume of tap-water consumption per day (number multiplied by glass size), minutes of showering/bathing per week (frequency × duration of bathing/showering), minutes per week spent washing dishes and clothes by hand without gloves, respectively (frequency × duration of each activity), and swimming pool attendance (yes/no) during pregnancy.

*Outcome data.* Basic information regarding the infants, including gestational age, sex, birth length, and birth weight, were collected from the clinical birth records. Gestational age was based on the interval between the last menstrual period and the date of delivery of the infant. Small for gestational age (SGA) was defined as a live-born infant below the 10th percentile of birth weight for gestational age in a national Chinese referent population (Chen and Jin 2011).

*Blood sample collection and blood THM analyses.* A 5-mL blood sample was collected by nurses ≥ 2 hr after last showering/bathing on the first day of hospital admittance of pregnant women waiting for delivery. After the blood draw, the tubes were shaken to dissolve the anticoagulant (potassium-ethylenediaminetetraacetic acid) immediately, kept in coolers and then shipped to the laboratory. The blood samples were kept at 4°C before they were analyzed for THMs within 2 weeks (Bonin et al. 2005).

Concentrations of THMs in blood samples were determined by headspace solid phase micro-extraction (SPME)–gas chromatography with an electron capture detector (GC/ECD; Agilent Technologies 6890 N). The detailed method and quality control have been described in our previous study (Zeng et al. 2013). Briefly, we sealed 3-mL blood samples in 10-mL headspace vials. We then heated (20°C) and agitated (300 rpm) samples using a magnetic stirrer to facilitate extraction of volatiles from the sample headspace onto an SPME fiber. After extraction, we immediately inserted the fiber into a hot GC inlet and maintained it for 3 min. We identified the four individual THMs according to retention times. Final quantification was based on procedural standard calibration curves. The limits of detection (LOD) for TCM, BDCM, DBCM, and TBM were 1.9, 0.5, 0.7, and 2.0 ng/L, respectively. Concentrations below the LOD were assigned with LOD divided by the square root of 2 for the analysis.

*Statistical methods.* The Predictive Analytics Suite Workstation (PASW) version 18.0 (IBM Corporation, Armonk, NY, USA) was used for the analysis. Descriptive statistics for demographics, birth outcomes, and maternal blood THMs were conducted. To compare differences of fetal growth measures and gestational age in all categorical variables, parametric and nonparametric methods were appropriately used to test statistical significance. In addition, Pearson correlation analysis was used to examine the association between maternal age and fetal growth measures. Br-THM concentration was defined as the sum of BDCM, DBCM, and TBM in blood. Total THM (TTHM) concentration was defined as the sum of TCM and Br-THMs in blood. Because the detectable percentage of blood BDCM, DBCM, and TBM concentrations is not high, we used a three-level variable to categorize participants into low-exposure (< LOD) and equally sized medium- and high-exposure groups. We divided maternal blood TCM, Br-THM, and TTHM concentrations into tertiles based on measured values (none was < LOD), and used the lowest level as the reference. We conducted tests for trend by treating the blood THMs as an ordinal categorical variable in regression models.

General linear models were applied to analyze the association between maternal blood THM level and fetal growth and gestational duration indices (birth weight, birth length, and gestational age). Logistic regression models were used to estimate odds ratios (ORs) and 95% confidence intervals (CIs) for SGA infants. Covariates were included in the final models for each fetal outcome based on biological and statistical considerations. For the statistical consideration, potential confounders [gestational age, prenatal body mass index (BMI), weight gain during pregnancy, infant’s sex, study city, education, and household income] were entered into the final multivariable model with *p*-value < 0.2 for unadjusted associations with fetal outcomes. Maternal age and parity were included in final models based on biological consideration because previous studies have suggested that they are predictors of fetal growth (da Silva 2012; Shah and Knowledge Synthesis Group on Determinants of LBW/PT Births 2010).

All regression models were adjusted for the following dichotomous variables: prenatal BMI (< 28/≥ 28 kg/m^2^), weight gain during pregnancy (< 15/≥ 15 kg), infant’s sex (male/female), parity (no child/≥ one child), and study city (Xiaogan/Wuhan). As a continuous variable, maternal age (squared, years^2^) was included in all regression models; gestational age (weeks) was entered only into the models for birth weight and birth length. As a categorical variable, education (less than primary school, junior and senior high school, college and above) was included in the models for birth weight, birth length, and SGA; household income (< 3,000, 3,000 to < 5,000, ≥ 5,000 Yuan) was included in the models for gestational age and SGA. Statistical significance was defined as a *p*-value < 0.05. And statistically suggestive was defined as a *p*-value < 0.10 (Zeng et al. 2013).

## Results

*Characteristics of the study population.* The demographic characteristics of mothers and their infants are summarized in Table 1. Of the 1,184 single gestation live births, 60 (5.1%) were classified as SGA. The mean (± SD) birth weight, birth length, and gestational age were 3,322 ± 404 g, 50 ± 1 cm, and 39 ± 5 weeks, respectively. Mean maternal age was 29 ± 5 years old. The majority of mothers had prenatal BMI < 28 kg/m^2^ (68.5%), gained > 15 kg during pregnancy (61.6%), reported drinking < 1,200 mL of water per day (55.8%), and did not swim during pregnancy (99.4%) or hand wash dishes (73.0%) or clothes (53.4%) without gloves during pregnancy.

**Table 1:** Maternal and neonatal infant characteristics in the study populations and according to birth outcome.*^a^*

Variable	Study population [*n* (%)]	SGA [*n* (%)]	Birth weight (g) (mean ± SD)	Birth length (cm) (mean ± SD)	Gestational age (weeks) (mean ± SD)
Total births	1,184 (100)	60 (5.1)	3321.8 ± 404.1	50.0 ± 1.0	38.7 ± 4.6
Season
Spring	393 (33.2)	14 (23.3)	3351.1 ± 400.0	50.1 ± 1.0	39.0 ± 1.1
Summer	306 (25.8)	16 (26.7)	3313.0 ± 407.7	50.0 ± 1.3	39.1 ± 1.3
Autumn	386 (32.6)	24 (40.0)	3312.3 ± 409.1	50.1 ± 0.8	39.1 ± 1.4
Winter	99 (8.4)	6 (10.0)	3269.4 ± 386.5	50.0 ± 1.0	38.9 ± 1.2
Study city
Xiaogan	426 (36.0)	32 (53.3)**	3291.2 ± 404.6*	49.9 ± 1.3**	39.2 ± 1.2**
Wuhan	758 (64.0)	28 (46.7)	3338.9 ± 403.0	50.1 ± 0.9	39.0 ± 1.3
Infant’s sex
Male	630 (53.2)	27 (45.0)	3357.0 ± 403.6**	50.2 ± 0.9**	39.0 ± 1.1*
Female	554 (46.8)	33 (55.0)	3282.9 ± 401.3	49.9 ± 1.2	39.1 ± 1.4
Weight gain during pregnancy (kg)
< 15	448 (38.9)	32 (55.2)*	3252.5 ± 396.7**	49.9 ± 1.1**	39.0 ± 1.3
≥ 15	705 (61.1)	26 (44.8)	3367.6 ± 404.1	50.1 ± 1.0	39.1 ± 1.2
Prenatal BMI (kg/m^2^)
< 28	811 (68.5)	46 (76.7)	3272.4 ± 369.1**	50.0 ± 1.0**	39.1 ± 1.3
≥ 28	373 (31.5)	14 (23.3)	3429.1 ± 453.5	50.2 ± 1.2	39.0 ± 1.2
Education
Less than primary school	47 (4.0)	2 (3.3)*	3273.3 ± 381.0	49.5 ± 1.8**	39.0 ± 1.1
Junior and senior high school	624 (52.7)	41 (68.4)	3300.3 ± 399.3	50.0 ± 1.0	39.1 ± 1.3
College and above	513 (43.3)	17 (28.3)	3352.3 ± 410.5	50.2 ± 0.9	39.0 ± 1.2
Household income, RMB, per month (Yuan)
< 3,000	594 (50.2)	35 (58.3)*	3304.5 ± 407.7	49.9 ± 1.2	39.2 ± 1.3
3,000 to < 5,000	370 (31.3)	17 (28.3)	3316.7 ± 382.9	50.1 ± 0.8	39.0 ± 1.2
≥ 5,000	220 (18.5)	8 (13.4)	3376.9 ± 404.1	50.2 ± 0.8	39.0 ± 1.2
Parity
No child	881 (74.4)	43 (71.7)	3316.6 ± 391.8	50.1 ± 1.0	39.1 ± 1.2
≥ 1 child	303 (25.6)	17 (28.3)	3336.9 ± 438.1	50.0 ± 1.3	39.0 ± 1.4
Use of boiled water
Yes	1,080 (91.2)	51 (85.0)	3324.7 ± 403.9	50.0 ± 1.1	39.0 ± 1.3
No	104 (8.8)	9 (15.0)	3289.8 ± 408.2	50.1 ± 0.7	39.2 ± 1.2
Use of filtered water
Yes	205 (17.4)	9 (15.0)	3294.4 ± 401.1	50.1 ± 1.0	39.1 ± 1.1
No	975 (82.6)	51 (85.0)	3327.4 ± 405.3	50.0 ± 1.1	39.1 ± 1.3
Tap-water consumption
< 1,200 mL/day	632 (55.8)	33 (55.0)	3294.4 ± 388.2	50.0 ± 1.1	39.2 ± 1.3**
≥ 1,200 mL/day	499 (44.2)	27 (45.0)	3349.7 ± 418.3	50.1 ± 1.0	39.0 ± 1.3
Swim
Yes	7 (0.6)	0 (0.0)	3057.7 ± 347.6	49.8 ± 1.0	38.3 ± 1.3
No	1,177 (99.4)	60 (100)	3323.1 ± 404.0	50.0 ± 1.0	39.1 ± 1.3
Time of showering/bathing
< 70 min/week	544 (48.1)	25 (46.3)	3322.4 ± 394.1	50.1 ± 0.9	39.1 ± 1.3
≥ 70 min/week	586 (51.9)	29 (53.7)	3323.3 ± 413.7	50.0 ± 1.1	39.0 ± 1.3
Time of dishwashing
0 min/week	795 (73.0)	48 (81.3)	3325.4 ± 412.3	50.1 ± 1.1	39.0 ± 1.6
< 35 min/week	115 (10.6)	4 (6.8)	3276.4 ± 383.8	50.1 ± 0.7	39.1 ± 1.4
≥ 35 min/week	179 (16.4)	7 (11.9)	3323.6 ± 383.6	49.9 ± 1.1	39.0 ± 1.3
Time of washing clothes
0 min/week	584 (53.4)	32 (56.2)	3333.7 ± 426.4	50.1 ± 1.1	39.0 ± 1.2
< 40 min/week	250 (22.9)	10 (17.5)	3322.9 ± 386.4	50.1 ± 0.9	39.0 ± 1.3
≥ 40 min/week	259 (23.7)	15 (26.3)	3301.5 ± 369.0	49.9 ± 1.0	39.1 ± 1.4
Maternal age (years) (mean ± SD)	28.7 ± 4.6	28.3 ± 5.4	28.7 ± 4.6	28.7 ± 4.6	28.7 ± 4.6**
RMB, renminbi. ^***a***^31 missing weight gain during pregnancy, 4 missing usage of filtered water, 53 missing tap water consumption, 54 missing time of showing/bathing, 95 missing time of washing dishes, 91 missing time of washing clothes. *p-Value < 0.05. **p-Value < 0.01 for overall difference of fetal outcomes in the categorical and continuous variables.					

Water-use activities were not significantly associated with birth outcomes, except for tap-water consumption and gestational age (mean, 39.2 ± 1.3 and 39.0 ± 1.3 weeks among mothers who reported drinking < 1,200 and ≥ 1,200 mL/day, respectively, *p* < 0.01) (Table 1). However, the association was only suggestive after adjusting confounding (–0.15 weeks shorter; 95% CI: –0.31, 0.01 for ≥ 1,200 vs. < 1,200 mL/day; *p*-value = 0.07).

*Maternal blood THM concentrations.* The distribution of maternal blood THM concentrations among the study participants is presented in Table 2. TCM was detected in 92.5% of the blood samples, whereas BDCM, DBCM, and TBM were found at a lower frequency, ranging from 22.6% to 57.4%. The geometric mean (median) concentrations of TCM, Br-THMs and TTHMs were 40.7 (50.7) ng/L, 5.3 (5.6) ng/L and 52.3 (57.7) ng/L, respectively.

**Table 2:** Distribution of maternal blood THM concentrations (ng/L) (*n* = 1,184).

Exposure variables^*a*^	Percent detected (95% CI)	Geometric mean (95% CI)	Median (95% CI)
TCM	92.5 (91.0, 94.0)	40.7 (38.0, 43.6)	50.7 (48.0, 53.0)
BDCM	57.4 (54.6, 60.2)	1.5 (1.4, 1.6)	2.5 (2.0, 2.9)
DBCM	33.5 (30.8, 36.2)	0.9 (0.9, 1.0)	0.5 (0.5, 0.5)
TBM	22.6 (20.2, 24.9)	1.6 (1.6, 1.6)	1.4 (1.4, 1.4)
Br-THMs^*b*^	—	5.3 (5.1, 5.5)	5.6 (5.2, 5.9)
TTHMs^*c*^	—	52.3 (49.8, 55.0)	57.7 (55.1, 59.9)
^***a***^The LODs for TCM, BDCM, DBCM, and TBM were 1.9, 0.5, 0.7, and 2.0 ng/L, respectively. When the concentration was below the LOD, it was replaced with LOD divided by the square root of 2. ^***b***^Br-THMs: sum of BDCM, DBCM, and TBM. ^***c***^TTHMs: sum of TCM and Br-THMs.			

*Maternal blood THMs and fetal growth.* Table 3 presents regression coefficients for fetal growth associated with categories of maternal blood THM concentrations. We found no statistically significant associations between maternal blood THM concentrations and gestational age. Maternal TTHM concentrations in the second and third tertiles (44.2–74.4 and > 74.4 ng/L, respectively) were associated with lower birth weight relative to the lowest tertile (< 44.2 ng/L), with estimated mean differences of –59.09 g (95% CI: –114.46, –3.71) and –60.88 g (95% CI: –116.18, –5.58), respectively (*p* for trend = 0.03). Additionally, there was a suggestive negative association between birth weight and TCM (–48.23 g; 95% CI: –103.64, 7.19 for the third vs. first tertile, *p* for trend = 0.08). BDCM and DBCM were negatively associated with length at birth, with estimated mean decreases of 0.15 cm (95% CI: –0.29, –0.01) and 0.20 cm (95% CI: –0.37, –0.04), respectively, for the highest versus lowest exposure groups (*p* for trend of 0.04 and 0.02, respectively).

**Table 3:** Regression coefficients [β (95% CI)] for fetal development associated with categories of maternal blood THM concentrations (*n *= 1,184).

Blood THMs categories (ng/L)	Birth weight (g)^*a*^	Birth length (cm)^*a*^	Gestational age (weeks)^*b*^
TCM
< 38.2	0	0	0
38.2–67.1	–25.90 (–81.9, 30.13)	–0.08 (–0.23, 0.07)	0.13 (–0.05, 0.30)
> 67.1	–48.23 (–103.64, 7.19)	0.04 (–0.11, 0.18)	0.15 (–0.03, 0.32)
*p* for trend	0.08	0.63	0.10
BDCM
< 0.5	0	0	0
0.5–4.8	–27.41 (–82.94, 28.11)	–0.03 (–0.18, 0.12)	–0.05 (–0.23, 0.12)
> 4.8	–36.32 (–91.22, 18.58)	–0.15 (–0.29, –0.01)*	0.01 (–0.16, 0.19)
*p* for trend	0.18	0.04	0.93
DBCM
< 0.7	0	0	0
0.7–2.6	4.66 (–59.46, 68.78)	–0.05 (–0.22, 0.12)	0.00 (–0.20, 0.20)
> 2.6	–4.98 (–66.97, 57.02)	–0.20 (–0.37, –0.04)**	0.04 (–0.15, 0.23)
*p* for trend	0.92	0.02	0.72
TBM
< 2.0	0	0	0
2.0–2.4	19.01 (–54.49, 92.51)	0.05 (–0.15, 0.24)	–0.11 (–0.34, 0.12)
> 2.4	–24.72 (–96.99, 47.55)	–0.06 (–0.25, 0.14)	–0.06 (–0.29, 0.17)
*p* for trend	0.66	0.72	0.44
Br-THMs
< 3.3	0	0	0
3.3–7.5	–12.99 (–69.35, 43.36)	0.00 (–0.15, 0.14)	–0.01 (–0.19, 0.17)
> 7.5	–25.53 (–81.21, 30.15)	–0.04 (–0.18, 0.11)	–0.08 (–0.25, 0.10)
*p* for trend	0.37	0.62	0.39
TTHMs
< 44.2	0	0	0
44.2–74.4	–59.09 (–114.46, –3.71)*	–0.10 (–0.25, 0.05)	0.06 (–0.11, 0.23)
> 74.4	–60.88 (–116.18, –5.58)*	0.00 (–0.15, 0.14)	0.14 (–0.04, 0.31)
*p* for trend	0.03	0.96	0.12
^***a***^Adjusted for gestational age, infant’s sex, maternal age, prenatal BMI, weight gain during pregnancy, education, parity, and study city. ^***b***^Adjusted for infant’s sex, maternal age, prenatal BMI, weight gain during pregnancy, household income, parity and study city. *p < 0.05. **p < 0.01.			

The ORs and 95% CIs for SGA and maternal blood THM concentrations are shown in Table 4. Exposure to Br-THMs was positively but only suggestively associated with SGA (OR = 1.48; 95% CI: 0.71, 3.04 and OR = 1.92; 95% CI: 0.98, 3.79 for the second and third exposure groups, respectively; *p* for trend = 0.06). SGA was significantly increased in association with the second and third tertiles of TTHMs (OR = 2.91; 95% CI: 1.32, 6.42 and OR = 2.25; 95% CI: 1.01, 5.03, respectively, *p* for trend = 0.08).

**Table 4:** ORs and 95% CIs for SGA with categories of maternal blood THM concentrations (*n* = 1,005).*^a^* ^,^ *^b^*

Blood THMs categories (ng/L)	SGA (*n*)	Adjusted OR (95% CI)
TCM
< 38.2	13	1
38.2–67.1	24	1.62 (0.78, 3.36)
> 67.1	23	1.48 (0.71, 3.08)
*p* for trend		0.32
BDCM
< 0.5	25	1
0.5–4.8	18	1.30 (0.65, 2.59)
> 4.8	17	1.15 (0.58, 2.28)
*p* for trend		0.66
DBCM
< 0.7	40	1
0.7–2.6	10	1.16 (0.54, 2.52)
> 2.6	10	1.18 (0.56, 2.49)
*p* for trend		0.62
TBM^*c*^
< 2.0	52	1
≥ 2.0	8	0.81 (0.33, 1.99)
*p*		0.65
Br-THMs
< 3.3	16	1
3.3–7.5	18	1.48 (0.71, 3.04)
> 7.5	26	1.92 (0.98, 3.79)
*p* for trend		0.06
TTHMs
< 44.2	9	1
44.2–74.4	28	2.91 (1.32, 6.42)**
> 74.4	23	2.25 (1.01, 5.03)*
*p* for trend		0.08
^***a***^Excluding 179 large for gestational age (LGA) based on weight, defined as a live-born infant above the 10th percentile of birth weight for gestational age in a Chinese national referent population. ^***b***^Adjusted for infant’s sex, maternal age, prenatal BMI, weight gain during pregnancy, education, household income, parity, and study city. ^***c***^TBM was divided into two groups by LOD due to the small number of SGA in high level. *p < 0.05. **p < 0.01.		

## Discussion

We determined the maternal blood THM concentrations as an internal dose level of THM exposure, which could represent an accurate and integrative measure of all routes and sources of exposure. Because blood concentrations are strongly influenced by very recent exposure, and showering and bathing have been shown to have a stronger influence on blood levels than other water-use activities (Nuckols et al. 2005), we collected blood samples after at least 2 hr since last showering/bathing to gain a relatively steady state of THMs in the blood. Two studies have shown that blood samples that were taken after 30 min since last showing/bathing can provide a window to a steady-state level (Ashley et al. 2005; Silva et al. 2013). Ashley et al. (2005) reported decrease in blood THM concentrations from 5 min to 30 min after shower/bath among 7 young and healthy subjects. Silva et al. (2013) also found that blood THM concentrations dropped rapidly during the first 30 min after showering among 100 study participants following a controlled showering exposure. Consistent with two previous reports, TCM was the main component of blood TTHMs in our study population (> 70%) (Miles et al. 2002; Riederer et al. 2014). The median concentrations of blood THM were higher than reported for a representative sample of U.S. adults (National Health and Nutrition Examination Survey participants in 1999–2006) (Riederer et al. 2014) and a group of 150 U.S. women (Rivera-Núñez et al. 2012), but were similar to levels reported for 401 men from Wuhan, China (Zeng et al. 2013).

TTHMs were associated with a significant decrease in mean birth weight, with similar estimated reductions for the second and third tertiles of exposure compared with the first. We also found some evidence for associations between exposure to TTHMs during late pregnancy and the risk of SGA, which was consistent with previous studies that reported a small increased risk of SGA for high-exposure TTHMs (Hinckley et al. 2005; Hoffman et al. 2008b; Porter et al. 2005; Wright et al. 2003, 2004). Many previous studies have characterized exposures based on total blood THM concentrations, though a few have evaluated exposures to individual THMs. For the individual THMs in our study, we found associations of reduced birth length with individual brominated THMs (e.g., BDCM, DBCM) but not with TCM. However, we found a suggestive association of reduced birth weight with TCM but not with individual brominated THMs; this was consistent with previous toxicological studies in rats showing that brominated THMs were more harmful to the fetus than TCM (Narotsky et al. 1997; Ruddick et al. 1983). Exposure to brominated THMs (e.g., BDCM, 50 and 75 mg/kg; DBCM, 200 mg/kg) can result in reduced body length and increased rate of fetal resorptions, whereas only exposure to higher doses of TCM (400 mg/kg) can result in reduced fetal body weight (Narotsky et al. 1997; Ruddick et al. 1983). Except for our study, only one other study (Patelarou et al. 2011) has reported an excess risk of SGA based on length (a live-born infant below the 10th percentile of birth length for gestational age in a referent population) for the higher tertile of brominated THMs (adjusted OR = 1.3; 95% CI: 0.5, 2.7). The association between THMs and birth length requires further investigation.

We did not find a statistically significant association between gestational age and THM exposure during pregnancy, which was also consistent with the results of several other studies, including a recent meta-analysis (Aggazzotti et al. 2004; Grellier et al. 2010; Jaakkola et al. 2001). However, others found that exposure to high TTHMs may prolong gestational duration and reduce the risk of preterm delivery (Hoffman et al. 2008a; Lewis et al. 2007; Wright et al. 2004). These inconsistent results may be attributed to differences in the characteristics of the study population, in exposure assessments, and in the ability to control for confounding factors.

Several limitations of our study should be mentioned. First, because the demographic profiles and the neonatal infant characteristics were different between our studied cities, we adjusted for potential risk factors for fetal growth that varied between cities to control for potential confounding between cities. Although some of the covariates (e.g., season, smoking, exposure to secondhand smoke, alcohol use, gravidity, maternal medical risk factors during pregnancy) varied between cities, they were not included in the final models because they did not predict fetal outcomes with *p* < 0.2 in bivariable models. Nevertheless, the study city was a predictor of gestational age. This suggests that there may be other unmeasured factors that affect gestational duration and vary by study city (Hoffman et al. 2008a). In addition, the detailed data on other potential confounders were missing, including prenatal care access, isolated maternal medical risk factors (e.g., chronic nephritic syndrome, anemia, uterine bleeding during pregnancy), and dietary habits (e.g., fasting), which might bias the relationships observed in our study. Furthermore, our previous study conducted in a water supply system in Wuhan has shown that THM levels in drinking water are below the regulatory limits of China (Zeng et al. 2014a). However, THM levels in the other water supply systems in Wuhan, as well as in Xiaogan, were missing. Thus, the contributions of water THMs to the internal dose of THMs and birth outcomes were unclear.

Second, we relied on a single blood sample during the third trimester to estimate exposure. Although it has been suggested that blood concentrations at a single point in time may reflect steady-state levels (Blount et al. 2011), high-exposure events such as showering, bathing, and swimming can have a substantial effect on blood concentrations (Nuckols et al. 2005; Silva et al. 2013). Changes in routine water-use activities in late pregnancy, and dietary changes (e.g., fasting) before delivery, also may cause blood THM concentrations to fluctuate, both within and between days (Ashley et al. 2005; Riederer et al. 2014). Thus, future studies should collect multiple blood samples to provide a more accurate measure of steady-state levels, and use longer-lived exposure biomarkers (e.g., protein or DNA adducts) to avoid this limitation (Blount et al. 2011).

Third, although the third trimester is the most important for fetal growth (Diamond 2001; Grellier et al. 2010), some studies have found an association of TLBW (term low birth weight) or SGA with high levels of TTHMs during the second trimester (Lewis et al. 2006; Wright et al. 2003), suggesting that exposure before the third trimester may also hamper fetal growth. Pharmacokinetic changes during pregnancy, including increases in plasma volume, changes in blood protein binding, and fat accumulation during the first two trimesters and increased CYPE1 and CYP2D6 activity in the third trimester also may influence the relationship between environmental exposures and resulting blood THM concentrations (Anderson 2005; Choi et al. 2013). Therefore, assessment of exposure with exposure biomarkers during different trimesters of pregnancy deserves attention in future studies.

Finally, we estimated associations between exposure to drinking-water DBPs and fetal growth based on blood THM concentrations. However, people are generally exposed to DBP mixtures in drinking water that may include other DBPs that are more toxic to fetal growth than THMs (Richardson et al. 2007). For example, previous studies have reported that SGA and decreased birth weight are associated with urine TCAA, a biomarker that reflects ingestion of DBPs in chlorinated drinking water (Costet et al. 2012; Zhou et al. 2012). Because the physicochemical properties, exposure route, metabolism, and toxicity among different DBP classes vary, THMs may not be a valid marker of exposure to other DBPs that may be more etiologically relevant (Zeng et al. 2014b). Thus, the associations of other specific DBPs should be considered in future studies.

## Conclusions

In the present study, we used whole-blood THMs as exposure biomarkers to estimate associations between exposure to THMs in drinking water and fetal growth outcomes and gestational age. We found that elevated maternal blood THM concentrations were associated with decreased birth weight, reduced birth length, and increased risk of SGA, suggesting that elevated maternal THM exposure during late pregnancy may adversely affect fetal growth. However, one-time blood THM concentrations may not be good biomarkers of DBP exposure in general during pregnancy. Further studies with different exposure biomarkers for trimester-specific exposure monitoring are needed.
